# Pilot study for the understanding and use of probiotics by different paediatric healthcare professionals working in different European countries

**DOI:** 10.1186/s13052-019-0648-4

**Published:** 2019-05-03

**Authors:** Massimo Pettoello-Mantovani, Fügen Çullu Çokuğraş, Mehmet Vural, Julije Mestrovic, Luigi Nigri, Ruggiero Piazzolla, Ida Giardino, Michele Conoscitore, Leyla Namazova-Baranova

**Affiliations:** 1 European Paediatric Association/Union of National European Paediatric Societies and Associations (EPA/UNEPSA), Berlin, Germany; 2 Department of Pediatrics, Scientific Institute “Casa Sollievo della Sofferenza,”, University of Foggia, Foggia, Italy; 3 Turkish Paediatric Association/University of Istanbul, Istanbul, Turkey; 4 National Paediatric Society of Croatia/Medical School of Split, University Hospital of Split, Split, Croatia; 5 Italian Federation of Paediatricians, Rome, Italy; 6 Union of Paediatricians of Russia/Russian Medical Research and Scientific medical University of Moscow, Russian Federation, Moscow, Russia

**Keywords:** Probiotics, Survey, Healthcare

## Abstract

**Background:**

Consumers’ conviction of the benefits of probiotics is influenced by their existing beliefs and by the information they receive from healthcare professionals. The attitude of healthcare professionals towards commercially available probiotics will, therefore, determine how trustworthy and beneficial these products are perceived by consumers. Furthermore, due to European Union legislation, companies are prohibited from displaying information on product packaging; therefore, consumers are dependent primarily on healthcare professionals for correct information and guidance on the use of these products. The aim of this pilot study was to explore the understanding and use of probiotics in clinical practice by professionals who are involved in child healthcare in different European countries and to assess how much they value the scientific evidence behind these products.

**Methods:**

The study was performed using a cross-sectional, descriptive, 30-question online questionnaire circulated among healthcare professionals belonging to three professional categories that are typically involved in childhood probiotic prescription: paediatricians, dieticians and general practitioners. The questionnaire was developed using web-based standard guidelines, and the questions were modelled on those used in previously published probiotics studies.

**Results:**

Overall, 27,287 healthcare professionals belonging to three major European scientific societies were contacted by the organizations participating in the study. In total, 1360 valid questionnaires were recorded, and the results were statistically analysed.

**Conclusions:**

The results emphasize the importance for healthcare professionals to be properly educated and updated on probiotics. An improved knowledge about probiotics led to increased prescriptive confidence. To disseminate accurate information on probiotics, healthcare professionals look for appropriate and scientifically validated educational platforms to acquire information, explore concerns and barriers and look for positive approaches towards recommending probiotics.

## Introduction

Probiotics [1–3] are currently prescribed for a variety of clinical conditions [4, 5] with which there is credible evidence to support their use [5–8]. Guidelines have been developed by the European Society for Paediatric Gastroenterology, Hepatology, and Nutrition (ESPGHAN) for the use of probiotics in the management of acute gastroenteritis [8, 9], and their use in the form of fermented products such as yogurt has been reported by ESPGHAN to prevent the occurrence of antibiotic-associated diarrhoea (AAD) [10]. Furthermore, meta-analyses [4, 11, 12] have shown probiotics to be effective in decreasing both the incidence and duration of common infectious diseases, such as upper respiratory tract infections, with a substantial impact on public health and socioeconomics [6]. Their beneficial use in clinical practice has been recently described in children for selected clinical conditions and in specific vulnerable groups [5]. However, the correct use of probiotics in clinical practice by healthcare professionals (HCPs) appears to be complicated by a limited knowledge of the scientific basis for their use and by the ample, yet confusing, information provided by industry on commercially available probiotic products. In general, primary care professionals do not rate their understanding of probiotics as very high, and they report being confused by the terminology used to describe the gut microbiological environment and the properties of probiotic-containing products, as in the case of live yogurts [13]. Consumers’ conviction of the benefits of probiotics is influenced by their existing beliefs and by the information they receive from HCPs [14]. The attitude of HCPs towards commercially available probiotics will therefore determine how trustworthy and beneficial these products are perceived by consumers [15]. Furthermore, European Union (EU) [7] legislation prohibits companies from including informational text on probiotic packaging; thus, consumers are dependent primarily on HCPs for correct information and guidance on probiotic use.

In Europe, the field of paediatrics is characterized by a large amount of diversity, variation, and heterogeneity in the healthcare that is provided to the more than 200 million children below 18 years of age within the 53 European countries. This implies a significant diversity in the availability of commercially prescribed products and in the available information and prescription attitudes regarding these products [16]. The aim of this pilot study was to investigate the levels of understanding and use of probiotics in the clinical practice of European professionals who are involved in paediatric healthcare who work in different European countries (characterized by diverse medical training backgrounds) and to evaluate how much HCPs value the evidence behind these probiotics. A further aim was to investigate which educational platforms are considered by HCPs to be useful for acquiring information and exploring concerns and barriers and for providing positive approaches towards recommending probiotics. The results of this preliminary study help to decide whether to further engage in similar investigations involving additional countries and different types of HCPs in order to develop useful recommendations on how to disseminate accurate information on probiotics to the population and overcome possible local cultural barriers and/or insufficient medical communication.

## Methods

This study was planned by the working group on nutrition of the Union of National European Paediatric Societies and Associations/European Paediatric Association (EPA/UNEPSA) and performed during July–October 2016. A questionnaire focusing on the understanding and attitude towards commercially available probiotic products was circulated among HCPs belonging to three professional categories that are typically involved in childhood probiotics prescription: paediatricians, dieticians and general practitioners.

### Design of the questionnaire

A cross-sectional, descriptive, 30 question online was developed and validated in 2016 at the University of Nottingham School of Biosciences within the frame of the Master of Science degree programme in Advanced Dietetic Practice. It was designed in DanSurvey, a licenced product of LimeSurvey (Symfony, England), which is a free and open source online survey application written in PHP, based on a MySQL, PostgreSQL or MSSQL database, and distributed under the GNU general public license [17].

The questionnaire was developed using web-based standard guidelines [18], and the questions were modelled on those used in previously published studies on probiotics [19]. Factors that may influence the choice of probiotics, including demographics (country of work, gender, profession), areas of expertise, the association between nutrition and clinical practice and the attitude towards probiotics, were considered in the choice of questions [20].

In accordance with previous studies, the questionnaire was designed to take less than 30 min to complete in order to reach an acceptable compliance and response rate [21]. The 30 questions were divided into three sections: *About you* (*n.6*), *Probiotics* (*n.21*) and *How to get information to you* (*n.3*). The questionnaire was based predominantly on multiple choice questions with an open field to clarify the answer where required.

### Survey participants and data collection

The Union of National European Paediatric Societies and Associations promoted the questionnaire among the 50 national European paediatric societies belonging to the organization. The national paediatric societies of Croatia, Italy, Turkey and Russia agreed to participate in the survey and circulated the questionnaire among their paediatrician members. In these four countries, only doctors certified in paediatrics are allowed to be involved in paediatric healthcare. Dieticians participating in the survey were from United Kingdom (UK), including members of the British Dietetic Association (BDA), which agreed to collaborate in the survey. Primary care gastroenterologists were also included in the study, as they are also involved in paediatric healthcare and are classified in the survey data analysis as general practitioners (GPs). The GPs were members of the European Society for Primary Care in Gastroenterology (ESPCG), a professional scientific society that also agreed to collaborate in the data collection by circulating the questionnaire among their European associates who are involved in paediatric care in hospital, community or primary care settings.

To facilitate data collection, the questionnaire was translated into the national language of the participating organizations (Croatian, Italian, Turkish and Russian). To ensure that the respondents accurately understood the authors’ intention, the translated questionnaires were validated by the paediatrics and statistics departments of the following institutions: the Scientific Centre of Children’s Health, Moscow, Russia; the University of Foggia, Italy; the University of Split Medical School, Croatia; and the University of Istanbul, Turkey. The participants were allowed to forward the link to the questionnaire to colleagues practising in European countries who were also involved in paediatric care and belonged to the same professional area (dieticians working in paediatric healthcare, paediatricians and GPs with expertise in paediatrics), thereby soliciting additional, completed questionnaires. This resulted in responses from countries in addition to the UK, Italy, Croatia, Turkey and Russia and is reported in the results section.

### Statistics

Frequency analyses were applied to check for data errors, and any values outside of this range were easily identified [22] and recoded to fit into existing categories, otherwise new categories were created to group the responses. Most of the data were described using univariate analysis. Chi-squared tests were used to compare the respondents. A significance level of *p* ≤ 0.05 was used. The ‘other please specify’ responses were subjected to content analysis, coded and added to the quantitative data.

SPSS software (version 23. 2015, IBM, USA) was used for statistical analysis, with a license obtained from Nottingham University for the purpose of this research. A convenience sample was used to compare the three groups (dietitians involved in paediatric care, GPs involved in paediatric care and paediatricians) [23].

The statistical analysis was validated at the University of Nottingham School of Biosciences in the UK within the frame of the Master of Science degree programme in Advanced Dietetic Practice and was further elaborated and confirmed by the statistical analysis unit of the European Association of Paediatrics and the University of Foggia Department of Paediatrics in Italy.

### Ethics

Ethical approval was received from the School of Biosciences Research Ethics Committee (SBREC150106A, 12/10/2015). The survey was anonymous, with no personal or identifiable data being collected. The study was approved by the Ethics Committee of the European Paediatric Association/Union of National European Paediatric Societies, Office of Presidency.

## Results

A total of 27,287 HCPs were contacted by the six scientific societies participating in the study, including 7000 dietitians contacted by BDA, 20,000 by the national paediatric societies of Croatia, Italy, Russia and Turkey, and 287 by ESPCG. In the study, 1604 responses were recorded; of these, 244 were removed due to insufficient data recorded on the forms, leaving 1360 valid questionnaires (Table 1). The highest absolute number of respondents (*n* = 846) were paediatricians, followed by dieticians (*n* = 426). Of the 287 GPs who received the questionnaire circulated by ESPCG among its members, 36 responded. GPs made up 2.6% of respondents, dietitians 31.3% (*n* = 426) and paediatricians 62.3% (*n* = 846). For the other 52 (3.8%) respondents, it was not possible to determine the profession, and their data were reported separately.

**Table 1:** Characteristics of survey respondents. Breakdown by profession, country of origin, gender and year of graduation

Country	Dietitian N (%)	Paediatrician N (%)	GP N (%)	Other N(%)	Total N (% of respondents)
United Kingdom	409 (95.6)	10 (2.2)	5 (13.9)	2 (3.9)	426 (31.6)
Russia	2 (0.5)	342 (40.2)	2 (5.5)	6 (11.5)	352 (26.1)
Italy	2 (0.9)	201 (23.5)	7 (19.5)	17 (32.7)	227 (16.9)
Turkey	0	223 (26.2)	0	1 (1.9)	224 (16.7)
Croatia	0	66 (7.6)	0	0	66 (4.8)
Other country	13 (3.0)	4 (0.3)	22 (61.1)	26 (50.0)	54 (3.9)
Total	426 (100)	846 (100)	36 (100)	52 (100)	1360 (100)
Gender					
Female	388 (93.3)	585 (70.2)	15 (42.8)	30 (57.7)	1018 (76.2)
Male	24 (5.8)	240 (28.8)	20 (57.2)	21 (42.3)	305 (22.8)
Prefer not to say	4 (0.9)	9 (1.0)	0	0	13 (1.0)
Total	416 (100)	834 (100)	35 (100)	52 (100)	1336 (100)
When graduated					
last 5 years	158 (37.1)	330 (39.1)	6 (17.2)	14 (26.9)	508 (37.4)
5–15 years ago	118 (27.7)	197 (23.2)	5 (14.3)	17 (32.8)	337 (24.9)
> 15 years ago	150 (35.2)	316 (37.3)	24 (68.5)	21 (40.3)	511 (37.6)
Did not declare	0	1 (0.4)	0	0	1 (0.1)
Total	426 (100)	844 (100)	35 (100)	52 (100)	1357

### Respondent profile

All healthcare professionals enrolled in the study worked in the field of child healthcare. Of those who declared their gender, more women (*n* = 1018, 76.2%) than men (*n* = 305, 22.8%) contributed to the study (Table 1). The highest total number of respondents were from the UK (*n* = 426, 31.6%), followed by Russia (*n* = 352, 26.1%), Italy (*n* = 227, 16.9%), Turkey (*n* = 224, 16.7%) and Croatia (*n* = 66, 4.8). The remainder of respondents (54, 3.9%) were from different European countries, including 19 from Greece (1.3%). The majority of respondent dietitians were from the UK, while the respondent paediatricians were variously distributed among the four European countries of the paediatric national societies that participated in the study. Only 14 paediatricians were from other European countries, of which 10 were from the UK (Table 1). The 36 respondent GPs were variously distributed among several European countries.

**Fig. 1:**
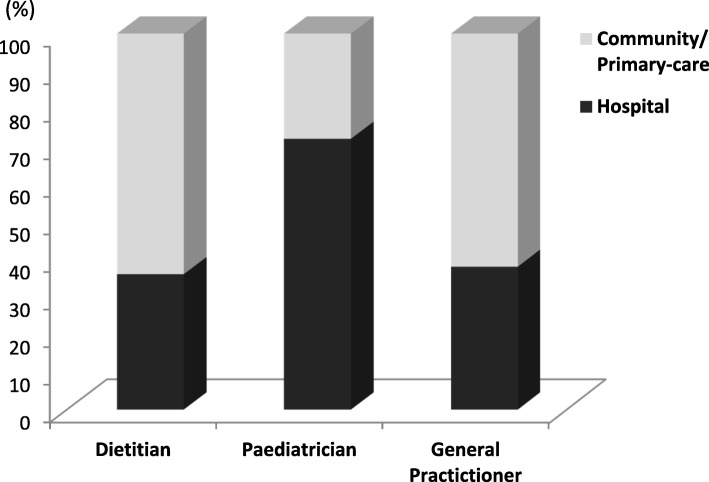
Paediatric healthcare setting of responders (in%)

The respondents worked in different healthcare settings [16], which are reported in Fig. 1.

Dietitians mainly worked in hospitals (*n* = 224), followed by the community (*n* = 199). Overall, most of the paediatricians were hospital-based (*n* = 611), while the rest worked in primary care settings (*n* = 235). Regarding professional qualifications, 1357 respondents completed this question. The majority qualified more than 5 years ago (Table 1). In particular, 337 (24.9%) received their professional qualifications between 5 and 15 years ago, while 511 (37.6%) qualified more than 15 years ago.

### Attitudes towards probiotics among different professions

Regarding the statement that nutritional advice plays an important role in clinical practice, there was a high level of agreement amongst the dietitians (*n* = 298, 83.2%), paediatricians (*n* = 753, 93.5%) and GPs (*n* = 24, 77.4%). Conversely, the dietitians also disagreed the most with this statement (*n* = 57, 15.9%), as shown in Table 2.

**Table 2:** Attitude of HCPs towards nutritional advice and probiotics

		Dietitian	Paediatrician	General Practitioner	other	did not declare	Total
Nutritional advice plays an important role in my clinical practice.	Disagree	57	34	3	0	1	95
		15.9%	4.2%	9.7%	0.0%	4.8%	7.7%
	Neutral	3	18	4	1	0	26
		.8%	2.2%	12.9%	5.6%	0.0%	2.1%
	Agree	298	753	24	17	20	1112
		83.2%	93.5%	77.4%	94.4%	95.2%	90.2%
Probiotics have a place in clinical medicine.	Disagree	14	47	5	0	0	66
		3.9%	5.8%	16.1%	0.0%	0.0%	5.3%
	Neutral	64	36	0	1	2	103
		17.9%	4.4%	0.0%	5.6%	9.5%	8.3%
	Agree	279	727	26	17	19	1068
		78.2%	89.8%	83.9%	94.4%	90.5%	86.3%
Probiotics are an evidence based intervention for health?	Disagree	34	38	3	0	2	77
		9.5%	4.7%	9.7%	0.0%	9.5%	6.2%
	Not sure	100	153	6	8	5	272
		28.0%	18.9%	19.4%	47.1%	23.8%	22.0%
	Agree	223	618	22	9	14	886
		62.5%	76.4%	71.0%	52.9%	66.7%	71.7%
How likely are you to suggest a probiotic food or drink?	Unlikely	98	103	7	4	5	217
		27.9%	12.8%	22.6%	23.5%	23.8%	17.7%
	Don’t know	59	60	1	2	3	125
		16.8%	7.5%	3.2%	11.8%	14.3%	10.2%
	Likely	194	641	23	11	13	882
		55.3%	79.7%	74.2%	64.7%	61.9%	72.1%

Of those who completed the question on whether probiotics have a place in clinical medicine, 86.3% (*n* = 1068) agreed. In contrast, 21.2% (*n* = 78) of the dietitians did not agree that probiotics had a place in clinical medicine. When respondents were asked whether they believed probiotics to be an evidence-based intervention for health, there was a higher level of agreement amongst paediatricians (76.4%, *n* = 618) and GPs (71%, *n* = 22), than dietitians (63.3%, *n* = 223). Dietitians who responded negatively totalled 37.5% (*n* = 134) as opposed to 23.6% (*n* = 191) of paediatricians and 29.1% (*n* = 9) of GPs.

Overall, HCPs are likely to suggest a probiotic food or drink (*n* = 882, 72.1%) in comparison to freeze-dried probiotics in tablet form. Dietitians (*n* = 157, 44.7%) were more likely to not recommend a probiotic food or drink (combined responses to ‘don’t know’ or ‘unlikely’), in comparison to 20% (*n* = 163) of paediatricians and 25% (*n* = 8) of GPs (Table 2). HCPs seem to be largely unaware that in Europe companies are legally prohibited from communicating about probiotics directly with consumers (*n* = 842, 78%).

### HCPs rating their knowledge of the gut microbiota

**Fig. 2:**
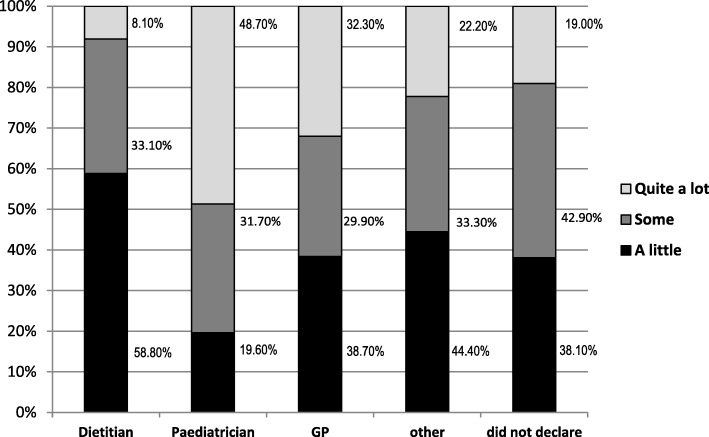
Level of training in probiotics reported by HCPs

When asked whether there is a need to educate HCPs about probiotics, 91% (*n* = 1120) of the respondents answered positively. The survey asked the respondents to rate their level of training in probiotics and their knowledge of the gut microbiota. When comparing the three professional groups, dietitians had the least training in probiotics, with 91.9% (*n* = 328) describing their training in probiotics as some/a little versus 51.3% (*n* = 416) of paediatricians (Fig. 2).

When assessing how HCPs rate their knowledge of gut microbiota/intestinal flora, 16.5% (*n* = 59) of dietitians rated their knowledge as quite high. This is in contrast to paediatricians, where 54.3% (*n* = 437) rated their knowledge as quite high (Table 3).

**Table 3:** How HCPs rate their knowledge of the gut microbiota

	Dietitian N (% of respondents)	Paediatrician N (% of respondents)	GP N (% of respondents)	Other N (% of respondents)
Quite a lot	59(16.50)	437(54.30)	15(48.40)	6(37.5)
Some	158(44.30)	279(34.70)	13(41.90)	5(31.3)
A little	140(39.20)	89(11.10)	3(9.70)	5(31.3)

**Fig. 3:**
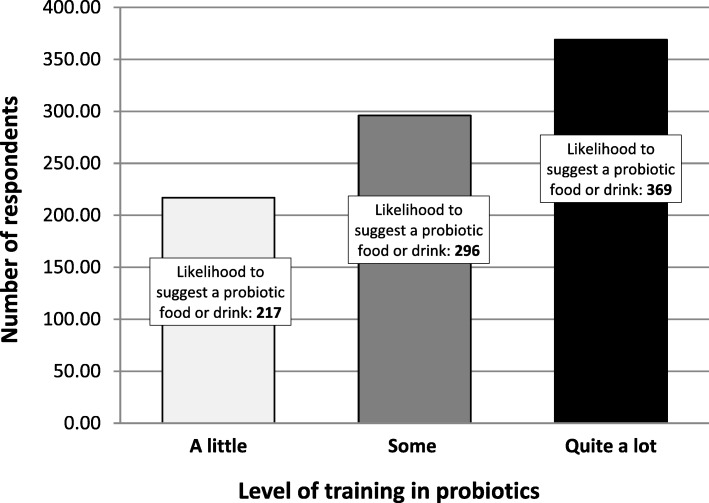
Level of training in probiotics cross-tabulated with the likelihood of HCPs to recommend a probiotic

Looking at how the level of training in probiotics correlates to the likelihood of recommending a probiotic food or drink, the data analysis indicates a moderate but significant positive correlation (*r* = 0.241) with the highest level of training (“quite a lot”). The chi-squared tests suggested that HCPs who had much training were more likely to recommend probiotics (*p* < 0.005) (Fig. 3).

### Health disorders indicated by HCPs to benefit from probiotic prescriptions

The patient groups that paediatricians and dietitians are most likely to recommend probiotics for are children with irregular bowel movements and diarrhoea (*n* = 705, 51.8%). Within this result, 72.9% (*n* = 617) of paediatricians, as opposed to 12.4% (*n* = 53) of dietitians, would recommend a probiotic for children with irregular bowel movement and diarrhoea. Pertaining to diarrhoea in young children, 73.8% (*n* = 624) of paediatricians, as opposed to 14.1% (*n* = 60) of dietitians and 47% (*n* = 17) of GPs, would recommend a probiotic. Among the dietitians, 48.4% (*n* = 206) would recommend a probiotic for bloating and 58.5% (*n* = 249) for irritable bowel syndrome (IBS) across the general population. For allergies in the general population, 42% (*n* = 355) of paediatricians, as opposed to 7.7% (*n* = 33) of dietitians and 11% (*n* = 4) of GPs, would recommend a probiotic.

### HCPs’ evaluation about the evidence supporting probiotics

The areas where the majority of HCPs (*n* = 957, 81.5%) agreed on the efficacy of probiotics include the role of probiotics in balancing the intestinal flora and enhancing its functionality. The majority of paediatricians confirmed their belief in the evidence behind AAD (*n* = 553, 71.1%). Overall, 67.6% of HCPs agreed with the evidence (*n* = 803, 67.6%) for AAD. Forty percent (*n* = 313) of paediatricians stated that probiotics alleviated thrush, compared to 18% (*n* = 63) of dietitians and 26.7% (*n* = 4) of GPs. The questions did not specify oral thrush. A large percentage of HCPs neither agreed nor disagreed with the statements that probiotics alleviate thrush (*n* = 512, 43.8%), allergic psoriasis (*n* = 696, 59.4%), improve infection control (*n* = 538, 45.6%) and reduce the risk of a light common cold (*n* = 520, 44.8%).

### Position of HCPs on probiotic information

**Fig. 4:**
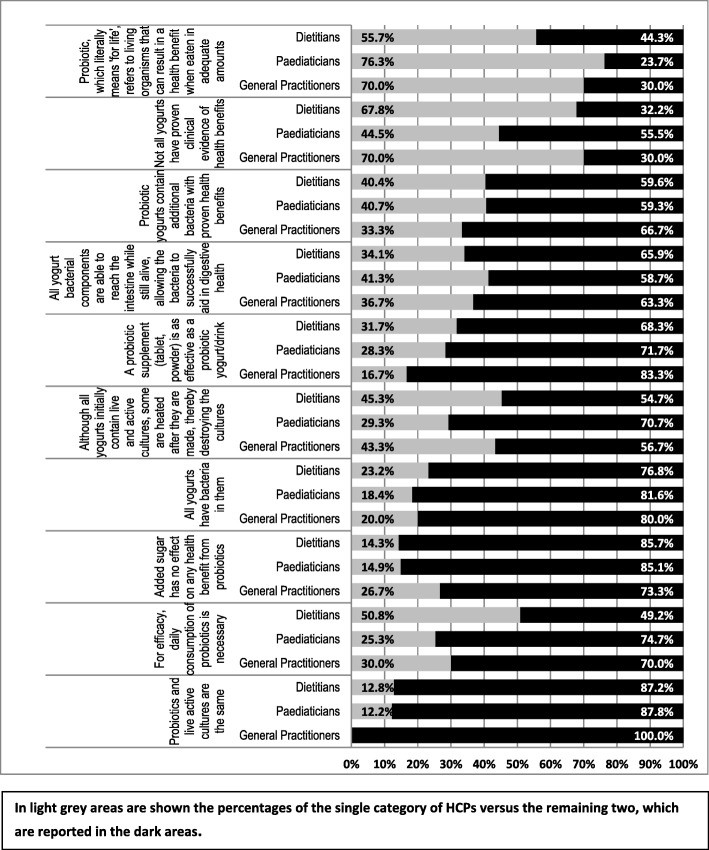
Statements about the use of probiotics. Percentage of statements that HCPs have agreed with

**Fig. 5:**
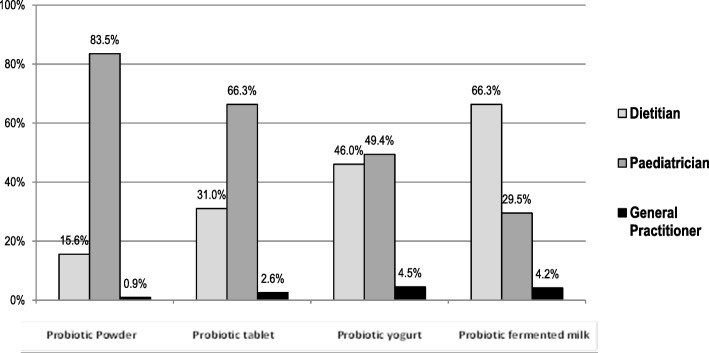
Formulation of probiotic HCPs prefer to recommend

The survey contained 10 statements on probiotics where respondents could select whether they agreed with the statement. Figure 4 summarizes the results by percentage of the responses from each of the HCP groups (dietitians, paediatricians and GPs). Over 76% (*n* = 482, 76.3%) of paediatricians were familiar with the World Health Organization (WHO) definition of probiotics [16], as opposed to 55.7% (*n* = 230) of dietitians. The majority of dietitians agreed that not all yogurts have proven clinical evidence of health benefits (67.8%, *n* = 280), as did 70% of GPs (*n* = 21), while 44.5% (*n* = 281) of paediatricians agreed with the statement. Figure 5 displays the probiotic formulation that HCPs participating to the study prefer to recommend.

Regarding whether probiotic yogurts contain additional bacteria with proven health benefits, 40.4% (*n* = 167) of dietitians and 40.7% (*n* = 257) of paediatricians agreed, as opposed to 33.3% (*n* = 10) GPs. Dietitians and paediatricians agreed that a tablet/powder would be as effective as a food (31.7% (*n* = 131) and 28.3% (*n* = 179), respectively).

### Areas of concern raised by HCPs

**Fig. 6:**
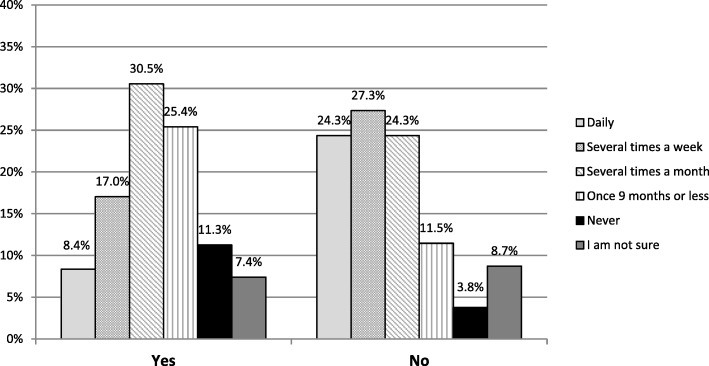
The cross-tabulation of recommendation of probiotics in practice according to concerns about their use

As dietitians responded more cautiously to whether they would recommend probiotics, it was worth further investigating their concerns. Their concerns were captured as free text and were then grouped into 5 areas. Table 4 shows the number of dietitians that raised these concerns. The most frequently cited concerns were of immunosuppressed patients and knowledge/education, followed closely by evidence/efficacy. Cost was also a concern raised by HCPs, although in a limited number of respondents. A chi-squared test showed how these concerns impact recommendation habits: the more concerns an HCP has, the less likely they are to regularly recommend a probiotic (*p* < 0.005) (Fig. 6).

**Table 4:** Areas of concerns for dietitians recommending probiotics

Concern	n (%)
Immunosuppressed patients	50 (28%)
Education	49 (28%)
Evidence/Efficacy	46 (26%)
Cost	19 (11%)
Other	12 (7%)
Total concerns raised	176

### Preferred mean for continuing education

HCPs prefer to obtain information at conferences (*n* = 995, 86.8%), workshops (*n* = 945, 82.6%), courses (*n* = 926, 81.3%) and study days (*n* = 795, 73%) rather than from social media sites, where 20.2% (*n* = 215) selected Facebook, 14.5% (*n* = 152) selected Twitter, 14% (*n* = 115) selected Linked In, 6.8% (*n* = 71) selected Instagram, and 5.2% (*n* = 54) selected Pinterest.

## Discussion

There is an increasing demand from the general population for credible information about probiotics and their use in clinical practice. In particular, HCPs are considered by children’s families and caregivers as the gatekeepers of dependable probiotic information. This questionnaire aimed at providing useful data able to better assist the development of proper responses to the request for viable information. The study investigated the attitudes towards probiotics of HCPs working in different European countries. The purpose of this study was to explore HCPs’ prescribing attitudes, counselling habits and knowledge of probiotics to obtain data that would be useful for developing proper, although generalized, recommendations for education on this topic, regardless of possible existing differences among the countries where the HCPs practice.

In general, the results of this study confirm that the knowledge and interest about the role of gut microbiota in health and disease has significantly increased in recent years, most likely due to the considerable number of high-quality studies and meta-analyses published in the scientific literature [24–26]. In fact, the majority of all HCPs in the study (86.3%) agreed that probiotics have a place in clinical medicine, and a significantly large number of them (72%) are likely to recommend a probiotic. These results were similar, with no significant differences in percentage of answers between the UK dietitians and the four groups of paediatricians working in the different countries (Croatia, Italy, Russia and Turkey), who are characterized by a diverse medical background and training in paediatrics [16].

However, data from the group of 409 dietitians working in UK indicate that this group of professionals do not agree to the same degree that probiotics are an evidence-based intervention for health. In fact, on further investigation, the data showed that only 55.3% of dietitians were likely to recommend a probiotic, although they believed probiotics have a place in clinical medicine (78.2%), and only 37% percent of dietitians reported being unsure or not convinced of the evidence. These findings show a lower percentage than those found in a previously published UK survey that was carried out predominantly amongst clinical practice nurses and among GPs and dietitians, where 91.2% of dietitians were likely to recommend a probiotic [13].

The questionnaire showed that among the three categories of HCPs participating in this study, paediatricians appeared to be more confident in their knowledge of probiotics and their ability to recommend a specific product, regardless of the country in which they practised. However, it is not possible to conclude whether this finding could be generalized or is related to local factors, such as medical background, practising habits or commercial information that could influence the prescription habits of the paediatricians working in the four participating countries. Furthermore, when asked to choose the statements they agreed with (out of a list of 10 statements), the group of dietitians working in the UK had a low level of awareness of the WHO definition of a probiotic, although it has been widely publicized. In contrast, paediatricians working in Croatia, Italy, Russia and Turkey and GPs practising in different European countries had a higher level of agreement on the definition of a probiotic (see Fig. 4).

The majority of HCPs were unaware that EU legislation prohibits manufacturers of probiotic-containing products from labelling their products and communicating about probiotics with consumers [27]. The results of this study emphasize the importance of HCPs to be properly educated [28] and updated on probiotics, as patients and families would like their HCPs to be informed and knowledgeable about using probiotics as a treatment option and need to feel comfortable in talking about the use of probiotics as part of complementary treatments [29]. The results of this survey also suggest that, in general, increased knowledge about probiotics leads HCPs to have increased confidence regarding these products [30]. Professionals who rated their knowledge as high were also more likely to recommend a probiotic. Finally, to acquire further information on probiotics, the majority of HCPs favoured more traditional forums, such as courses, workshops, conferences and study sessions.

## Conclusions

The purpose of this study was to contribute to a better understanding of probiotics in the clinical practice of European professionals who are involved in paediatric healthcare. However, the existing diversity in paediatric healthcare systems and the diverse medical context between European countries are limiting factors for studies aiming at developing standardized clinical practice or medical education programmes. Indeed, there are huge variations in Europe in the delivery of healthcare services, prescribing habits and medical counselling for children and families [16]. For instance, there are significant differences among European countries in the organization of paediatric hospital and nonhospital first-contact services. These services are provided in various forms among the 53 European countries, and they depend on whether primary care GPs, primary care paediatricians or a combination are primarily responsible for care and whether other HCPs, such as nurses or dieticians, are involved instead of paediatricians in certain areas of clinical care, including nutrition.

These factors have a general impact on studies involving different European medical contexts and may account for the intrinsic limitations of this study, such as the development of comparable conclusions between the data obtained from different countries. However, although it is not possible to draw conclusive evidence or develop generalized guidelines or protocols based on the results of this study, the data generated here offer practical, helpful information that may contribute to the development of useful recommendations for local European contexts.

In particular, the results of this study suggest that educational institutions, scientific organizations and policymakers should develop programmes to provide HCPs and their professional bodies with up-to-date, validated scientific content as training materials. Training programmes need to address basic and targeted health concerns, including information on what a probiotic is, which strains are present in commercial products, which health disorders could benefit from the use of probiotics, and finally, what the appropriate dosages are.

Educational institutions, scientific organizations and policymakers could play key roles to develop scientifically accurate and evidence-based educational content on probiotics. As probiotics are often commercially produced, industry could provide effective information platforms to better circulate correct and scientifically validated information to the public.

Finally, the findings of this pilot study suggest that this type of investigation should be expanded, and specific comparisons between groups of HCPs based on the country of work, the area of expertise and the clinical field should be carefully explored. Further studies would enable the acquisition of valuable information to help develop recommendations for the use of probiotics in paediatric clinical practice.

## Abbreviations

AAD: Antibiotic Associated Diarrhoea
BDA: British Dietetic Association
EPA/UNEPSA: Union of National European Paediatric Societies and Associations/European Paediatric Association
ESPCG: European Society for Primary Care in Gastroenterology
ESPGHAN: European Society for Pediatric Gastroenterology, Hepatology, and Nutrition
EU: European Union
GPs: General Practictioners
HCPs: Healthcare Professionals
IBS: Irritable Bowel Syndrome
UK: United Kingdom
WHO: World Health Organization
